# Addressing health inequalities in times of austerity: implementation of a place-based approach in multitiered local government

**DOI:** 10.1177/17579139241241194

**Published:** 2024-04-26

**Authors:** C Lee, M Buswell, J Coker, S Buckner, A Cowan, M Lhussier

**Affiliations:** Cambridge Public Health, Interdisciplinary Research Centre, Department of Engineering, University of Cambridge, and Cambridge Institute for Sustainability Leadership, Trumpington Street, Cambridge, CB2 1PZ, UK; Cambridge Public Health, Interdisciplinary Research Centre, University of Cambridge, Cambridge, UK; Cambridge Public Health, Interdisciplinary Research Centre, University of Cambridge, Cambridge, UK; Cambridge Public Health, Interdisciplinary Research Centre, University of Cambridge, Cambridge, UK; Cambridge Public Health, Interdisciplinary Research Centre, University of Cambridge, Cambridge, UK; Director, Centre for Health and Social Equity (CHASE), Northumbria University, Newcastle upon Tyne, UK

**Keywords:** place-based, asset-based, realist theory, inequalities, system-change

## Abstract

**Aims:**

This article focuses on how local authorities in England are tackling wider determinants of health and inequalities in their population’s outcomes while budgets for public services are diminishing.

**Methods:**

It reports the experience from one case study engaged in rolling out a devolved, place- and asset-based strategy over multiple tiers of local government. Relating these findings to relevant social theory, we draw out aspects of context and mechanisms of change. We offer plausible hypotheses for the experiences observed, which supports transferability and implementation of place-based strategies in other local authority areas struggling with similar challenges.

**Results:**

Findings highlight the importance of high-level and political buy-in, as well as the role of the COVID-19 pandemic as a potential catalyst to rollout. Creating the foundations for a new, place-based working was important for achieving coherence among partners around what local government was trying to achieve. These included investment in infrastructure, both relational and tangible inputs such as organisational and human resources, to establish the conditions for systemic change towards early intervention and prevention.

**Conclusion:**

This study identified clear foundations for place-based action, plus enablers and barriers to significant transformation of practice towards asset-based approaches between local authorities, partners and the public.

## Introduction

Many English Local Authorities (LAs) have adopted place-based strategies to tackle wider determinants and health inequalities in recent years. Several factors limit the potential for effective action, particularly limited resources, with LAs operating under increased financial constraints and uncertainty,^1–3^ compounded by lack of evidence on the most effective allocation of resources.

A resilience-building strategy (henceforth the Strategy) is strongly grounded in asset-based approaches (ABAs), including Asset-Based Community Development (ABCD),^4,5^ whose rationale includes the potential to mediate spatial inequalities. Several have sprung up across England (and globally) in recent years.^6–8^ ABAs take a ‘strengths-based’ rather than ‘deficit’ approach to health, building on resources available within local communities, with central concepts including the empowerment of marginalised groups, community capacity, connectedness, and social capital.^9–11^ Nonetheless, as popularity has grown, they have also come in for criticism, particularly for a lack of attention to power and equity.^12,13^

This article aims to share insights on how one LA developed a place-based strategy to tackling poverty and health inequalities, since in the United Kingdom ‘Most LAs are embracing community-centred ways of working’.^14^ It recognises the legitimacy of such criticisms as well as the importance of scholarship and reflective practice in resolving implementation weaknesses, to bring about meaningful change within a context of constrained public finances.^14–16^ The Strategy began in 2018 as a partnership between component upper tier and 5 lower tier LAs to transform service delivery towards objectives of being: people-centred, place-based and solutions-focussed,^17^ and comprised the following key elements: decentralising and building place-based partnership working; focusing on community engagement; identifying assets and local priorities; investing in new staff roles; working closely with communities; and supporting local action.

The estimated population was 653,537 (2019), characterised by a slightly higher proportion of older adults aged over 65 and ethnically less diverse than the national average (92.6% white vs 85.4%). Geographical ‘pockets’ consistently had considerably poorer health and wellbeing outcomes than the majority of the LA population, sometimes worse also than national averages, most notably a largely rural District and the main city centres, aligning with priorities for tackling spatial health inequalities.

## Aims and Objectives

This was a realist-informed study of the early implementation of the strategy. The study began in late 2019 and continued ‘light touch’ during the global pandemic (2020), picked up in 2021 (pandemic recovery), and completed in early 2022.^18^ At this time, key concepts had been introduced to link across, and to better connect, the upper and lower tiers. Some community engagement had been initiated and a core team of place-based staff were being recruited (one coordinator and up to two ‘community connectors’ per District). When the COVID-19 pandemic hit, the strategy workforce was redeployed to outbreak management and support to communities. The study sought to make the theory and change mechanisms underpinning the pathway between strategy implementation and impact on wider determinants of health explicit, while drawing out evidence of change, contextual barriers and enablers, emergent practice, and transferable learning.

The questions we aimed to answer were as follows:

1. How does this place-based strategy intend to address public heath priorities while operating within constrained budgets? (What is the underlying hypothesis?)
2. What can we learn from those professionally engaged in the strategy about the mechanisms of change to support future implementation and generate positive public health outcomes?

## Methods

**Figure 1:**
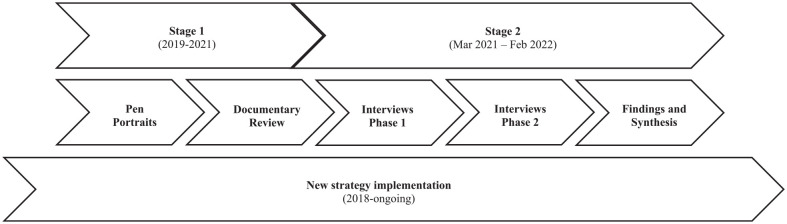
Study process

Two stages of data collection took place (Figure 1):

1. Review of documentary evidence and population health data.
2. Interviews with key stakeholders.

Stage 1 focused on the origins of the strategy, uncovering the underlying theoretical basis adopted, plus key features of context in terms of population health, wider determinants and inequalities. The resulting overview steered selection of informants for stage 2 and guides for discussion. Stage 2 data collection began at the strategic level of LA decision-making and followed its implementation over the study period.

### Fieldwork

From April 2021 to February 2022, 15 online interviews were recorded with the LA’s strategic leads and strategy staff, followed by staff working directly in local communities for other organisations.

### Analysis

In keeping with realist analysis, we developed and refined programme theories detailing Context, Mechanism (resource and response), Outcome configurations.^19^ Applying Wiltshire’s approach to identifying data themes,^20^ we examined the change pathways and mechanisms leveraged through the strategy, additionally identifying barriers and facilitators in the operating context.^21^ Documentary analysis focused on identifying Initial Programme Theories (IPTs), that is initial thoughts, formulated in programme theories, about how the strategy may be leading to desired outcomes in this particular context. Those were tested and refined through the phase 2 interviews. Interviews were deductively coded to a Nvivo framework intended to test and refine the IPTs developed in phase 1, followed by inductive reasoning to generate alternative or more nuanced explanations. We then identified plausible connections and theories with greater explanatory potential to support learning and transferability.^21^ Five interviews were double-coded, with all interviews sense-checked according to a sample of coding nodes.

## Results

### Foundations for change

Interviews indicated an evolution of place-based working over time, involving LA staff with ‘community’ portfolios, community-engagement teams and public health, in different community-focused pilots.^22,23^ Concurrent to the strategy rollout, 21 Primary Care Networks (PCNs) and 7 Integrated Neighbourhood Teams (bringing together health, social and community care professionals) were also developing to facilitate the development of locally responsive integrated care to address wider health and wellbeing issues.

A consensus emerged among participants that the LA needed to change the way it worked, against a backdrop of inequalities, growing need among residents, and financial constraints threatening service cuts and rising thresholds of access to care. The LA narrative also reflected ideas of enabling people to do more for themselves. A central tenet was the move to a more proactive approach, enabling the support of people ‘prethreshold’ before they need more costly services. One LA participant described the challenge as finding,

*‘the most appropriate and most local way of delivering services and addressing social immobility and inequality’* (Interviewee 1).

This change was primarily represented by a shift from an acute, demand-driven approach to service provision to a decentralised, placed-based *preventive* approach, focusing on the strengths and ‘assets’ present in communities. While the strategy articulated broad outcomes, such as ‘resilience’, and service avoidance or delay, specific population health goals were largely absent, although broad priority areas were later identified (e.g. food security, older adult services).

### A strategy of resilience building

**Figure 2:**
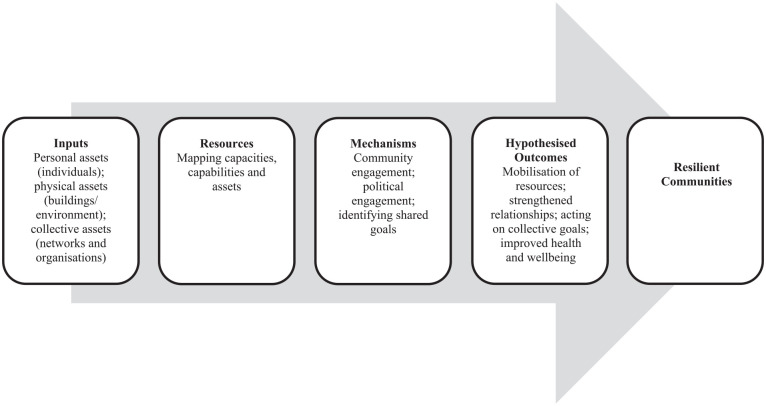
Theory of change for ABCD and improvement of public health outcomes

Outcomes typically anticipated through adoption of ABAs are thought to arise as a result of better mobilisation of assets and community engagement, as illustrated in the theory of change (Figure 2) adapted from Blickem et al.^24^

The Strategy was insufficiently mature to capture even intermediate population health outcomes within the study period, just as the ‘Wigan Deal’ and ‘Compassionate Frome’ models that inspired it only started reporting public health impact a decade after their introduction.^25–27^ Participants expected this,

*You really need a decade, especially when you are trying to do work that’s around prevention and trying to show that actually if you do things well at a community and place-based level then people won’t need help [. . .] it’s going to come big and bold in a decade or two decades or three decades.* (Interviewee 3)

That said, strong themes emerged that reflected important aspects of upstream organisational change and partnership working: triggers in the operating context; shared narrative; new ways of working, joint working and relational infrastructure; trust and relationships; physical and operational infrastructure. We explore these themes below, illustrating the process of shifting to place-based action on health and wellbeing and translation of theory into practice alongside evidence of any intermediate outcomes associated with introducing the Strategy.

### Enablers or ‘triggers’ in the operating context

The Strategy gained momentum during COVID-19 which catalysed its implementation countywide. ‘*Covid was the thing that turbocharged that way of working*’ (Interviewee 1). The Strategy was indeed effectively adopted as the blueprint for localised response during the pandemic. An example of this was local ‘COVID hubs’, established to coordinate place-based support to vulnerable community members: ‘*It’s helped the organisation and our partners recognise the value of taking a very place-based, person-centred approach to delivery. We’ve almost embedded [the Strategy] in our pandemic response*’ (Interviewee 4).

Senior LA management interviewees described the Strategy as a hopeful vehicle for ‘systemic change’, but one that prior to the pandemic had been difficult for key personnel to envisage. Being able to demonstrate its worth was highly valued by those charged with implementation: ‘. . . *trying to explain to people what we actually do is quite hard and we talk about using your local assets, using your strengths, making the connections, working together or whatever. People don’t always understand the value in that*’ (Interviewee 7). As such, the LA’s pandemic response seemed to provide a ‘win’ for the Strategy, building trust between the LA, partners and residents. The LA was able to publicise several examples of its resident partnerships minimising COVID’s negative impacts (e.g. at a conference of lowest tier local government and third sector partners).

Opportunities for local groups to ‘test out’ ideas to meet locally identified challenges was attributed to the relaxing of criteria for small grant funding during this time, overcoming scepticism that the Strategy was simply a ‘cost-cutting exercise’: ‘*We were more responsive and there was money that could be accessed relatively easily without going through 25 million procedures*’ (Interviewee 3).

While the speed of the ‘COVID response’ rollout offered benefits, there were drawbacks too, not least concerns that the Strategy was conflated with it:

*. . . I think we have gone a bit kind of cart before horse in a way. Because Covid happened and so then we kind of were doing [the Strategy] or doing what that looked like without actually having travelled the journey with people.* (Interviewee 1)

### Shared narrative

We identified a shared narrative around needing to shift from deficit-based descriptions of communities to one of community strengths, empowering residents to mobilise assets and build resilience:

*We definitely have a brand in [name of LA] (which is . . .) And that kind of puts the hooks for people, residents into ‘What is all this [the Strategy] malarkey then, what does it mean for us on the ground?’* (Interviewee 1)

Stakeholders across the system agreed with the intent to work in an ‘asset-based way’. Yet, although interviewees described features of ABCD as core Strategy components, *beyond* top tier LA staff there was little consensus around terminology. Three narratives emerged among the middle tier (local authority) and ground-level (non-LA) staff: the Strategy is: (1) well understood and familiar; (2) a catchy new label or shorthand for ‘*old-fashioned* *community development*’ and (3) lacks meaning, especially among non-statutory community workers and community groups.

Nevertheless, consultations with delivery partners helped them understand the aims and processes of the Strategy, to theoretically get people ‘on-board’:

*The key is that partners on the ground know what [the Strategy] is . . . There’s probably 50% of people that I talk to as partners, so other organisations, Local Authorities, charities, third sector partners that would understand what [the Strategy] is.* (Interviewee 12)

Another commented: ‘*I think that [the Strategy] as an approach has evolved to a point where it is no longer owned by one organisation*’ (Interviewee 1).

However, a senior leader reflected that although the LA Strategy ‘team’ had it ‘*completely embedded in our psyche, the way we operate, the way we talk*’ (Interviewee 15), it was not fully embedded organisationally, perhaps an unexpected consequence of prioritising engagement with external partners. Another concern was that it would become solely associated with the team whose roles were ‘badged’ as part of the Strategy.

### New relational infrastructure

The Strategy was described by interviewees as a way of working that united people, creating a *network* of people and support. It was also had an *enabling* role, hearing where the ‘energy’ was in communities and working with that, marking a change in the *culture* of the LA, from service delivery to facilitation, connection and coordination. Buyin at the highest political and administrative level was seen as significant to change:

*What we have got now is complete political backing from the leader of both [name of city and area local governments] and also from the Chief Executive down through Corporate Executive and Service Directors, which is something that we haven’t had before.* (Interviewee 13)

This new way of working bridged different tiers of local government and communities. The introduction of a ‘Place Coordinator’ role alongside ‘Community Connectors’ for each (lower tier local government) area was critical. One described their responsibilities as ‘*Going at this from a completely different angle … Bringing partners together at quite an organisational level, … service delivery level, we tend to be working with our five primary care networks in the area*’ (Interviewee 2).

This was complemented by the grass roots engagement function: ‘*I’ve got two engagement workers which … get involved in that nitty-gritty detail and feed up the chain to make those changes on the ground*’ (Interviewee 8). The narrative of ‘bringing people together’, ‘building bridges’ and ‘fostering relationships’ was consistent throughout the data.

The success of these roles was not taken as given, however, and we identify critical mechanisms of change as including taking *time* to discover, understand, learn, to establish *trust* and intervene where of value, not for the sake of it:

*My role is to go out there and meet people . . . And to start with I think it’s building up the trust as well, getting to know community leaders, community centres, etc., find out what they’re doing . . . Trust is really important.* (Interviewee 7)

*Role modelling* by LA staff, for example, from tackling ‘what is wrong’ in communities to identifying strengths and actions to build on, was seen as important: ‘*I suppose to try and help us to not work in the same way (as we always did) from a county perspective, but also to lead on the practical application of [the Strategy]*’ (Interviewee 1).

Some non-statutory sector community workers suggested some duplication of roles had emerged, however, with cross-sectoral engagement not always running as deep as intended: ‘*I would like to see … some of the county council’s senior staff who are managing this project come and talk to the other organisations that are out here every day doing what they do … Because nobody ever asks us*’ (Interviewee 14).

Complimentary interaction with what already existed nevertheless emerged as a strong theme among LA staff. Several accounts reported informal ‘asset-mapping’ by the Community Connectors alongside residents or community groups, as well as LA staff encouraging residents to work together and build on identified assets – fundamental elements of ABCD in practice.

### Trust and relationships

The importance of trust and relationships was a key theme: ‘*Community development is a long-term process of building trust and relationships*’ (Interviewee 11). An initiative seen as a precursor to the Strategy was cited to illustrate how relationships had improved over time:

*This is when it all started, we were keen to do things for our community on a health and well-being side full stop, and [name of initiative] was the first thing on that. And we had a big community meeting . . . so we could all work together, but they didn’t see it like that . . . 10 years down the line the two particular people I remember walking out are actually members of the [name of initiative] now* (Interviewee 5).

Informal communications were influential: ‘*A lot of that happens away from the meetings, it’s just emails, conversations, walking around together … things kind of trickle out of it, but we’ve formed those relationships, people know the other people working in the area*’ (Interviewee 2).

The creation of specific roles, such as Community Connectors, may be crucial to building social capital and capacity: ‘*And to start with I think it’s building up the trust as well, getting to know kind of community leaders, community centres, etc., … because it’s a small city everyone kind of knows everybody else*’ (Interviewee 7).

Nonetheless, a legacy of distrust in local government still festered in some areas, with lingering scepticism whether the Strategy’s public consultations had *really* incorporated community concerns: ‘*It comes across as a tick-box exercise so it can be said that community groups were spoken to when, in reality, the issues that matter to the community groups were not taken into account*’ (Interviewee 4).

There was also wariness among some community group leaders, who cited examples of historic community initiatives (e.g. skill-based youth services, funding for local voluntary/community groups), which were not sustained because of budget cuts. Any optimism about empowering community initiatives was tempered by such experience of past disappointments: ‘*There was an improving neighbourhood scheme in X and … then the government changed and that whole team was disbanded … it’s frustrating … the emphasis needs to be on sustainability*’ (Interviewee 8).

Indeed, there was a recognition that progress made did not guarantee the embedding of the Strategy, and fear that some relational gains would be lost postpandemic. One interviewee suggested operations were ‘slowly slipping back’ to pre-pandemic modes.

### Physical and operational infrastructure

Libraries were considered ‘physical assets’ representing locally anchored organisations that offer opportunities for residents to meet and access resources. Similarly, the concurrent use of physical buildings for problem-solving and collective action alongside ‘virtual’ spaces (during COVID lockdowns) was pragmatic solutions to shifting to place-based action.

Stakeholders were further engaged by the establishment of multiagency working groups to be rapidly deployed as required. While an LA-wide group predated the pandemic, some supportive local networks developed as part of the COVID response were seen as a ‘vital space’ for people to come together (possibly helping to build trust):

*One of the things that was set up very early . . . is our Local Connect Group . . . in the start of the pandemic it was meeting, I think, weekly . . . it normally has about 40 or so agencies represented . . . It brings together different parts of the public sector, our community faith leaders, voluntary sector leaders, various people from all over, adult social care colleagues.* (Interviewee 4)

A variety of small grant funding sources for local groups were cited as key to success – some time-limited opportunities launched during the pandemic, others more established programmes of community funding: ‘*We work with the community, we run the [name of project] and that supports new community groups … In the last five years we’ve supported 104 new community groups to get started*’ (Interviewee 14).

One programme of small grant funding is closely aligned with the Strategy’s intent to stimulate local action. An interviewee saw this as vital to building capacity in a locality otherwise deemed under-resourced:

*The only thing that we have responsibility for within the [locality primary care grouping] from an integrated neighbourhood point of view is the [name of fund]. So we’ve had two years of some money to develop innovation . . . we know that [locality name] CVS [Community and Voluntary Sector] doesn’t get the same kind of resources as . . . other organisations do.* (Interviewee 9)

While the Strategy was also able to offer small support, their ultimate goal was still seen by some as to discourage reliance on the statutory sector and public funding. It was ‘*more about helping people in the community to access sources of funds, but also use their own resources a lot more, either individually or by coming together, by making connections*’ (Interviewee 7).

A key contextual factor is the recent intensity of housing development across the area, including a ‘new town’. Such activity can generate funding at local government’s lowest tier, with developers required to partially repay the community to mitigate against negative impacts. In some cases, local government harnessed that funding to build community facilities:

*So that’s why [name of neighbourhood] is a bit different, because it does have this big pot of money that it can spend on community-led action, it can do some preventative and intervention work and it’s absolutely unique and amazing to have that much money to do this sort of thing.* (Interviewee 2)

Yet this meant there was no level playing field between new and established communities: ‘Then it would be more of a case of using the goodwill that’s within the community itself’ (Interviewee 2).

## Discussion

This study sought to understand how an LA place-based strategy responded to increasingly constrained budgets. The key components of ABAs are collaborative and partnership working, through which community strengths and resources are identified, developed and mobilised so that communities can be more self-supporting, avoiding more costly interventions.

Much evidence around the mobilisation of resources was understandably dominated by responding to a global pandemic. Nonetheless, clear themes likely to be strongly influential to the Strategy’s implementation under ‘normal’ circumstances were still apparent. These are presented in the form of realist programme theories in Table 1.

**Table 1:** Programme theory areas and component programme theories.

Programme theory area	Component PTa	Component PTb
**PT 1: Area wide transformation to focus on prevention.** In a context of increasing demand and limited resources, the LA introduces a strategy (resource mechanism) focused on building local strengths to foster system resilience (response mechanism) to increase self-reliance and reduce dependence on services (outcome)	**Investment in organisational infrastructure** The activation of investment in a new strategy (context) via creation of new roles at the District and community level (resource mechanism) formalised the focus on community assets (response mechanism) to facilitate the engagement and mobilisation of different actors and their resources to act collectively towards a common goal (outcome)	**Importance of high level buyin** In a context of collaborative working, senior level buyin and commitment from LA (resource mechanism) not yet replicated amongst other key system partners (response mechanism) is limited in ability to facilitate collaborative working (outcome)
**PT2: Place-based working** In a context of limited resources and increasing demand, a shared vision for partners coming together, pooling resources and working together (resource mechanism), means gaps can be minimised, duplication avoided and preventive work developed (response mechanism) to support local people to stay well and be supported when needed, further building community capacity (outcome)	**Shared focus and narrative** In a context of emergency response (pandemic context), shared focus between services (resource mechanism) enabled the aligning of priorities thanks to a sense of trust and common aims (response mechanism), resulting in meeting of urgent population need (outcome 1) and heightened understanding of community need (outcome 2)	**Importance of relational infrastructure** In localities with some foundations for place-based working (context), the time dedicated through the creation of new roles (resource mechanism) facilitates further connections to be made (response mechanism) supporting new ways of working (outcome)
**PT3: Cultural shift** A commitment to change at high level (context) enabled the Strategy (resource mechanism) to develop a shared vision (response mechanism), giving staff the impetus to best meet needs within their localities (outcome)	**New ways of working** The formalisation and widely shared commitment to a place-based strategy (context) enabled an investment in infrastructure (resource mechanism) facilitating innovative working on the ground (response mechanism), resulting in the development of bespoke place-based solutions (outcome)	**Collaboration** The infrastructure investment unlocked by the strategy (context) enabled the creation of new roles (resource mechanism), which are complimentary to other, pre-existing roles (response mechanism) in order to foster effective collaboration (outcome)

PT: Programme theory; LA: Local Authority.

Several interviewees highlighted the importance of senior-level commitment and significant resource investments in the Strategy (Table 1 PT1, CPTa and b). These include innovations described in PT3, CPTa: new roles and physical/virtual spaces to connect, communicate strategic rationale and share knowledge about resources and communities. According to Dart,^28^ all moves towards engaging communities are indications of systemic change.

There are warning signs, however, shown by our observations of unexpected outcomes. One explanation suggests that where new roles are perceived as unnecessary duplication of existing posts (in another sector), the expected response is not activated, perhaps due to inattention to context, generating negative responses among community partners (PT3, CPTb). Similarly, there was lack of coherence about understanding of the Strategy across the patch, possibly due to its conflation with the pandemic response. Any lack of ‘brand’ understanding could cause difficulties in mobilising partners to act collectively.

Another flag is raised if investment is only committed to short-term to staff who act as enablers and facilitators of support and services. If they are indeed the ‘glue’ that brings the system’s different parts together, they may also be foundational in the long-term. The shared experiences of short-termism in initiatives and funding underlines the importance of not fuelling any further disappointment or mistrust among community partners and reinforcing power imbalances.

Broader sociological theories associated with ABCD such as social capital, social networks, reciprocity, mutual aid,^29–31^ and community resilience were clearly associated with the Strategy.^32^ Hence, the ‘difference made’ by the Strategy was commonly articulated in ways allied to concepts associated with theory, rather than those easily quantifiable as ‘public health outcomes’ (e.g. increased collaborative working, community capacity, stronger relationships, trust). However, this lack of ‘hard’ evidence for reduction of health inequalities rather reinforces the criticism of ABAs as driven by austerity policies rather than true commitment to community empowerment – their promotion actively masking inherent power imbalance between relatively richer and poorer groups in society.^12,14^

Normalisation Process Theory (NPT) offered additional explanatory power when applied to the themes identified from our analysis.^33^ Mechanisms central to building the coherence that is a vital first stage in NPT can be seen as strongly related to factors associated with establishing better partnership working at system level. The ‘relational architecture’ identified (PT2) provided important resources for generating trust and shared values, vital to addressing need and tailoring local responses. Mechanisms such as a shared agenda, joined-up working and investment in community capacity were also deemed important enablers to place-based working in the Clear Horizons framework.^28^ Our findings and analysis equally resonate with a recent realist study of health alliances, delivering joint goals with a ‘whole system’ focus.^34^

In terms of context, we highlight the importance of what Mancini and Bowen^32^ term ‘community antecedents’ for developing community resilience. The legacy of joint working in one particular locality within the LA was supported by both significant time and resource investment (PT2). The theme of a ‘new way of working’ often recurred, drawing parallels with the ‘cognitive participation’ stage of NPT and the ‘cultural shift’ considered a key mechanism in transforming service delivery (PT3).^35^ The shared vision, values and development of trusted relationships are representative of this culture change, in turn facilitating ‘collective action’ and timely decision-making. Analysis of Community-Based Participatory Research (CBPR) partnerships supports this finding.^36^

Although there has been a ‘ripple effect’ of trust among system partners that may contribute to ‘sustaining collaborative efforts towards health improvement’,^36^ the relational infrastructure alone is insufficient to embed the strategy. Tailored investment that responds to existing resources, as well as gaps in context and community antecedents, is likely to be essential to underpinning new ways of working and truly empowering communities. Evidence on local contextual characteristics of importance are specifically lacking in the current evidence base on place-based approaches.^37^

The importance of this will remain as long as devolution remains significant to health and public service delivery. Our analysis suggests that insufficient attention is currently paid to the diverse contexts apparent across a large and varied patch in terms of geography, built environment, socio-economic profile and historical investment. Yet the fact that different neighbourhoods may have different starting points is emphasised by Cassetti et al’.s^35^ three AB models and our PTs (Table 1).

Aligning with emergent use of NPT in realist analyses,^38^ we formulate a new middle range theory to support programme implementation and transferability of our findings, as follows: within an operating context where joined-up working was already favoured (context: community antecedents), the development of a shared narrative around the value of community assets (resource mechanism: NPT coherence) led to increased trust and building of relational as well as operational infrastructure (response mechanism: NPT collective action) leading to greater community engagement and support for grass root action (outcome). Observation of a growth in grass roots action can be seen as a valid interim outcome to building community resilience, leading to decreased reliance on services.

## Conclusion

ABAs should not be adopted unquestioningly as the answer to health inequalities. Implementation context differs across geographies, and the insights developed here highlighting key points of context and change mechanisms are of practical relevance to implementing place-based approaches to public health in times of restrictive budgets. This study identified clear foundations (engagement, specific roles, shared values) for place-based action, stronger in some localities than others, plus enablers (building trust, small grant funding, less red tape) and barriers (conflation with COVID response, duplication of roles, contractual short-termism) to significant transformation of practice towards ABAs between LAs, partners and publics.
